# Surgical and Behavioral Relationships With Welfare

**DOI:** 10.3389/fvets.2020.00519

**Published:** 2020-08-14

**Authors:** Melissa Bain

**Affiliations:** Clinical Animal Behavior Service, Davis School of Veterinary Medicine, University of California, Davis, Davis, CA, United States

**Keywords:** animal, behavior, surgery, human-animal bond, medical, ethics, welfare

## Abstract

Veterinarians perform surgery for a number of reasons, from treating a problem to preventing future problems. There is an inextricable link between the physical and psychological aspects of an animal's health, and surgery is often a conduit to bridge that gap. Some surgical procedures can affect an animal's behavior, such as castration, and some pose an ethical dilemma, such as ear cropping and declawing. Ameliorating pain, decreasing stressful experiences for the animal, and identifying and treating concurrent problem behaviors are hallmarks of improving animal welfare. The purpose of this article is to outline some of these interrelationships and ethical dilemmas, providing evidence-based verification as applicable.

## Introduction

Problem behaviors are often cited as reasons for relinquishment and euthanasia of companion animals (1, 2). While not often thought to correlate with behavior, behavioral effects of surgery are commonly seen, but sometimes not fully understood. Surgery is a principal aspect of veterinary medicine, whether performed to treat a medical condition, to lower the incidence of disease, or for population control, and it is important to understand the impact that surgery has on the welfare and behavior of an animal. The effects on the behavior and, subsequently on the human-animal bond and welfare, should be taken into consideration when consulting with clients, either directly via the outcomes from the surgery, or via changes to the animal's behavior.

## History

John Hunter (1728–1793) is credited as the founding surgeon in human medicine. Discoveries at this time helped advance this area of medical care, with a primary focus on treating battlefield trauma, progressing to the treatment of other medical problems unrelated to trauma. In the veterinary medicine, however, advancements were not quite so quick. Until the 1920s, veterinary surgery was mostly limited to neutering and wound repair. As an example, aseptic technique was not utilized in large animal surgery until after World War II.

To demonstrate the rising importance of surgery in veterinary medicine, the American College of Veterinary Surgeons (ACVS) was founded in 1965 (3). The ACVS is a specialty board recognized by the American Veterinary Medical Association (AVMA) Boards of Veterinary Specialties and sets the standards for advanced professionalism in veterinary surgery. Although there is a higher level of training for specialists, all veterinarians are legally allowed to perform surgery. It's uncertain what percentage of all surgeries are performed by specialists compared to those performed by veterinarians in general practice or in conjunction with shelter, rescue, or other organizations. Given the greater number of veterinarians in general practice, and that veterinarians performing up to 45–50 surgeries per day in shelters, it is likely that non-specialists perform more total surgeries (4).

Understanding the inter-relationship between medical and behavioral problems in animals has been a point of discussion for a number of years. Some of the earliest documentation of this relationship is in the area of feline inappropriate urination (5–7). One of the earliest review articles of medical differentials for problem behaviors was published in 2003 (8).

## Surgery Performed for Underlying Problem Behaviors

Some surgeries are performed as emergency procedures related to an underlying problem behavior. Examples include surgery to treat a cat presenting for being hit by a car (transitioned to an outside cat due to inappropriate elimination in the house), a dog requiring multiple extractions of broken teeth from escaping its crate (due to separation anxiety), or a dog requiring a gastrotomy to remove rocks (due to pica). Others are performed to prevent or “treat” problem behaviors.

Emotions often run high for clients when going to a veterinary clinic for an emergency, as frequently they must make a hurried decision about the healthcare of their pet. Effective communication doesn't need to take more time, but perhaps takes a more concerted effort to overcome potential limitations of not having a long-lasting relationship with a client (9). It takes time for veterinarians and their staff to become culturally competent to most effectively gather information, as well as be comfortable asking open-ended questions to more efficiently gather information. Informed consent plays a role in all aspects of veterinary surgery, whether emergency or elective (10). It is necessary, however, to be able to obtain consent in a more truncated fashion in an emergency, where shared decision making may not be at the forefront of the process. Training in successful communication has received more attention in veterinary schools as of late, and is listed as highly sought after by veterinary employers (11).

These skills provide veterinarians with tools to investigate how problem behaviors may play a role in the presenting complaint, a skill in which veterinarians currently lack experience (12, 13). While it is important to provide urgent care, once the animal is stabilized, veterinarians should investigate the underlying reasons for the presentation. Although an individual veterinarian, such as a veterinary surgeon or one who is working in emergency and critical medicine, may not directly address the underlying behavioral reason for surgery, they should acknowledge the existence and offer resources for referral or treatment. These resources can include referral back to the pet's primary veterinarian or to a veterinary behaviorist.

## Peri-Operative Pain Management

Surgery, by design, involves incisions and tissue manipulation. These interventions cause discomfort at best, and intractable pain at worst. Veterinarians are increasingly aware of the need to identify and treat pain, but are often presented with challenges, sometimes brought about by their underlying attitudes (14–16). There are even some misconceptions on how different dog breeds perceive pain (17). Aside from the concerns surrounding ameliorating pain, one must also consider the role that pain plays in problem behaviors (18).

Identifying pain in animals is a growing competency. Whereas previously it was thought that some pain post-op helps keep the animal quiet, thankfully these beliefs are changing. There are different checklists and criteria by which veterinarians can measure pain in animals (19). Even with these measurements, there is sometimes a lack of agreement between veterinarians when evaluating levels of pain (20, 21).

Control of pain, whether related to an underlying physiological condition or due to post-operative complications, can be attained via a number of methods, and include nonsteroidal medications, opioids, and local anesthetics, along with other means such as physical therapy. Pain management is the subject of an increasing number of research studies and subsequent books and book chapters (22–24).

## Peri-Operative Behavioral Management

Behavioral management pre- and post-operative is also an integral part of decreasing stress surrounding these procedures. Many studies have demonstrated that animals exhibit stress-related behaviors and undergo physiological changes related to visiting a veterinary clinic (25–28). A study of dogs undergoing elective spay and neuter surgeries, with appropriate pain control, resulted in decreased interactive and exploratory behaviors, increased cortisol levels, short-term lymphopenia and eosinopenia, and long-term neutrophilia and monocytosis (29). It is uncertain whether these results were related to incomplete pain control or other stressors.

It is imperative that all staff in veterinary practices use appropriate means to alleviate anxiety, aside from pain control. These can include low-stress handling methods, playing music, and suitable housing. This is an increasing area of research, and many resources are available by which staff can learn and practice these techniques (30–37). Effective pharmacological interventions include trazodone, gabapentin, and transmucosal dexmedetomidine, as well as the use of pheromones (26, 38–42).

Kenneling animals in a veterinary clinic can be another source of stress. Kennels should be configured with the animal's physical and psychological comfort in mind whenever possible. One should remove collars and harnesses prior to placing in the kennel, as they can be choking hazards. If a cat is in a carrier, the open carrier can be placed inside the kennel until the cat exits the carrier, leaving the kennel in there as a hiding place for the cat.

All signs on the cage and notes in the medical record should be heeded, and the animal's behavior upon approach to the kennel should be assessed. Depending on the level of waste, etc., kennels should only be spot-cleaned during a patient's stay, and its “furniture” placed back in the same locations as the animal moved them. Of course, the kennel should be sanitized between patients or if the level of mess is at a critical level. The same towel and litterbox should be placed back into the cage if they are not dirty, to maintain a familiar scent. Animals should be provided with a place in which to hide or hide behind, such as a box for a cat or a towel covering the kennel door for all species, aside from those that require 24 h per day observations.

Environment outside of the cage should also be managed. Animals are sensitive to noise levels and can hear sounds outside of our level of hearing. Keep noise levels to a minimum (<60 dB—quiet conversational level), and keep dogs away from cats. Also, be sensitive when opening and closing cage doors as the metal clanging is jarring. Music can be played to help block out other sounds, but should be played at a quiet level. Studies show a potential for classical music to have a calming effect on animals (33, 43–45). Cats prefer a much warmer environment than what we prefer, between 35 and 38°C. Offer cats large towels so that they can burrow underneath, if desired, to help them maintain their preferred ambient temperature.

Staff should educate clients on how best to perform specific medical procedures required at home, both to prevent problems from occurring, as well as to help owners perform procedures when necessary. As examples, if owners are taught how to clean out their dog's ears, or how to give medications safely and effectively, they will be more likely to give their pet appropriate medical care. Depending on the medical or surgical procedure performed, different handling techniques should be highlighted, such as wound care, bandage changing, physical therapy, and bladder expression. Overarching all of this is proper pain control and medical management; if something is painful, no amount of behavior modification or low-stress handling will help the animal tolerate procedures.

Crate training prior to surgery can dramatically improve a dog's ability to cope with post-surgical confinement. Most dog owners have heard of crate training, but may not know how to effectively implement it. It takes time, effort, and patience, but when used properly, it can be a positive experience for both the owner and their dog. A dog cannot be expected to tolerate being in a crate post-operatively if it had never been trained prior, and if placed into a crate without behavior modification ca exacerbate its anxiety (46). Resources are widely available, including in veterinary behavior textbooks (47–49).

While it is ideal owners follow the recommendation for 100% confinement post-operatively, this likely will not occur, and they should be given explicit advice on how to interact with their pet. Such instructions can include the proper use of a sling to help a dog walk, safe ways to carry a pet, and what collar and leash to use. They should be given assorted ideas on how to safely provide for low-activity mental exercises, including food puzzle and toys and training exercises, such as the Protocol for Relaxation^1^ (50).

## Behavioral Treatment of Underlying Problem Behaviors

It is critical to address the underlying problem behaviors associated with the condition for which an animal requires medical care. Many textbooks provide veterinarians with detailed information on diagnosing and treating a myriad of problems (47, 48, 51).

As is the case in all areas of veterinary medicine, it is important to have a correct diagnosis in order to properly treat behavior problems (52). In one study of urine marking cats, almost a third of veterinarians did not meet the criteria of correctly diagnosing urine marking. Of those that did not mention the classic symptom of vertical urine marking, only 10% said that they had success in treatment (53).

Treating problem behaviors can be summarized in a “Five Step Treatment Plan.” These steps include management, relationship building, behavior modification (including desensitization and counterconditioning), tools, and pharmaceutical and other therapeutic treatments. While listed separately, they often overlap one another when one develops a treatment plan (54).

Management includes avoiding the trigger that causes the animal to either be placed into an undesirable emotional state, such as anxiety or fear, or display the unwanted behavior. Each time that the animal is in this situation it is then fearful or anxious. It also allows the animal to “practice” the behavior. An example of management is covering the front windows to prevent a dog from seeing passersby, thus, decreasing its barking behavior. Avoidance is also a safety recommendation, as a dog cannot be placed into a situation where it feels that it “needs” to bite someone.

Relationship building is a broad category covering educating the owner on an animal's emotional state (and the behavioral signs that signal such states), proper training techniques, and utilizing cues to teach an alternative behavior in a predictable and humane way. Some parts of this step can include: “Say Please” in which an animal is asked to perform a cue before a desired reward; and teaching cues that one can use to redirect in the future, such as “look” or “target.” A significant portion of this step may be spent refocusing the owner from using harsh punishment-based training techniques that can instill fear and anxiety in animals which increases the likelihood of a problem behavior surfacing.

Behavior modification is primarily focused on systematic desensitization and counterconditioning (DS/CC) toward the trigger that causes the animal to feel distressed and display unwanted behaviors (55). This is a process in which a conditioned emotional response (CER) such as fear is extinguished by exposing the animal in a graduated manner to the fear-eliciting stimuli, and replaced with an alternative, competing response by pairing it with an eliciting stimulus that will trigger an opposing emotional or physiologic response. When creating a program for systematic DS/CC, a stimulus hierarchy is created which ranges from a level that elicits no discernable response to a level that elicits an extreme response. The animal is exposed to the first step in this hierarchy, where it shows a very mild response, such as noticing that the trigger is present, until the mild response is no longer being displayed. Once this occurs, the animal is exposed to the next step on the stimulus hierarchy. All the while the animal is presented with something that will change the emotional response to one that is favorable toward the trigger, such as a high-value treat.

For example, a show cat may need to be bathed and dried prior to a show. If the cat is afraid of the sound of the blow dryer, the sound of the dryer can be paired with a favored food, which elicits pleasure. It is even more beneficial if the cat never gets this favorite food unless it is going through desensitization and counterconditioning. Counterconditioning occurs only if the new eliciting stimulus triggers a response powerful enough to supersede the original CER. If the cat is extremely afraid of the sound of the dryer, it is very likely that it will not eat in the presence of the blow dryer. By minimizing the intensity of the original conditioned stimulus, the new eliciting stimulus is likely to be salient enough to overcome it.

Tools are such products that are used to help implement management, relationship building, and behavior modification. Such tools include: muzzles, baby gates, and hiding places for cats (for management); clickers, treats, and toys (for relationship building); and leashes, harnesses, and treats (for behavior modification).

Pharmaceuticals and other therapeutic treatments are often needed to treat underlying anxiety that is hindering the effectiveness of behavior modification. Medications by themselves are not the answer, similar to how antibiotics are themselves not the answer for treatment of a cat bite abscess. Foundations of pharmaceutical interventions include quick-to-onset anti-anxiety medications, such as benzodiazepines, trazodone, gabapentin, and clonidine, and longer-to-onset medications, such as selective serotonin reuptake inhibitors (SSRIs) and tricyclic antidepressants (TCAs). Other therapeutics include pheromones, nutraceuticals, and special diets (56, 57).

There are specific behavioral treatments for the disorders for which surgical treatments have been suggested. Management, behavior modification, and perhaps the use of psychoactive compounds are the hallmarks of treatment for aggression, urine marking, barking, and scratching behaviors or, more specifically, the underlying reasons for these problems (58–60).

## Elective Surgical Procedures and Animal Behavior

### Castration and Ovariectomy/Ovariohysterectomy

Americans have been enculturated over the past number of years that one must neuter companion animals to prevent unwanted litters. The current estimate is that a high percentage of dogs in the United States are neutered and, even though there are recent shifts toward later neutering, it is often performed prepubertally, prior to 6 months of age (61). A survey of veterinarians in New York state demonstrated that 70% of veterinarians routinely recommend neutering for all dogs and cats, while roughly 35% believed that client-owned animals should not be neutered until at least 6 months of age (62). There are different attitudes toward neutering in other countries. As an example, roughly 15% of veterinarians in the U.K. believe that pre-pubertal neutering is not desirable, compared to roughly 8% of veterinarians in Australia and New Zealand (63). Reasons veterinarians give for neutering, especially at a young age, include the prevention of mammary and prostate cancers in females and males, respectively, that it prevents or helps decrease the chance of problem behaviors in dogs, and that it decreases pet overpopulation. The AVMA has a policy supporting pediatric spay and neuter of dogs and cats to reduce the number of unwanted animals, stating “*veterinarians should use their best professional judgment based on the current scientific literature in deciding at what age spay/neuter should be performed on individual animals*”(64). This in contrast to the British Small Animal Veterinary Association, which while recommending it for population control, states “*Before neutering for reasons of undesirable behavior it is important to consult a veterinary surgeon or animal behaviorist to ascertain the role of sexual hormones in the development and maintenance of the behavior* (65).

There is conflicting research on the potential positive, as well as harmful, outcomes from neutering. Veterinarians frequently cite the prevention of mammary gland cancer as a reason to spay dogs. However, a recent meta-analysis of published studies found that the link between ovariectomy in females and mammary cancer is weak at best (66). The link between castration and prostate cancer in dogs is even weaker, with some research showing an increased risk of this cancer in castrated dogs (67, 68). Aside from these medical conditions, more research is emerging on the potential negative effects of these surgeries. Increases in other types of cancer (osteosarcoma, hemangiosarcoma, lymphosarcoma, and mast cell tumors), orthopedic disease, immune-related disorders, and urinary incontinence are reported (69–77).

Sexually dimorphic behaviors are defined as those predominantly seen in one sex or another, and many are associated with reproduction or mate attraction. These behaviors are more strongly affected by castration. Castration usually abolishes overt sexual behavior in males, except for the more experienced the male, the longer mating behaviors persist (78).

The effect of castration on cat behavior can be quite dramatic. Fighting, roaming, and spraying are markedly reduced or eliminated in 80–90% of cats, usually with a rapid decline (79). There is no evidence of a relationship between age at time of castration and persistence of these behaviors, and neutered cats can still display these behaviors. Of castrated male cats, 10% are reported to be problem urine markers, while 5% of spayed female cats are reported to be problem urine markers (80).

Castration's positive effects on dog behavior are not to the extent seen in cats. Most improvement is seen in urine marking, mounting, and roaming behaviors, all of which are predominantly sexually dimorphic behaviors. There are additional behavioral reasons for these behaviors. As with cats, urine marking can be a symptom of an underlying anxiety-related disorder. Unrelated to sexual behaviors, mounting behaviors can continue as way of social communication or in a display of hyper-arousal in castrated dogs. Roaming behavior, which is related to mate-seeking behavior, can also be due to escape behavior, a symptom of separation anxiety in dogs. As with cats, there is no evidence of relationship between age at time of castration and persistence of these behaviors (81).

There is conflicting evidence on the effect of neutering on aggression in dogs. One paper demonstrated there was a decrease in aggression toward people and dogs after castration (81). However, another study found castrated dogs were no less likely to demonstrate aggression (82). Results from a paper evaluating the effects of gonadectomy in Viszlas were that the younger the age at gonadectomy for males and females, the earlier the mean age of diagnosis of a behavioral disorder (83). Results from another paper concluded that there was an increase in the odds of aggression toward unfamiliar people for all gonadectomized dogs compared with intact dogs. They determined this effect primarily seen in dogs gonadectomized at 7–12 months of age (82).

Ovariectomy usually abolishes sexual behavior in females. Alternatively, there is evidence that ovariectomy is correlated with an increase in aggression. One study demonstrated that, if a dog already displayed aggression as a juvenile, if spayed at <1 year of age, there was an increased chance of aggressive behaviors (84). The results of another study showed increased reactivity in spayed German Shepherd Dogs compared to those that were intact (85).

One must differentiate an ethical choice to neuter for pet population control, from a welfare choice for positively, or negatively, affecting an individual animal's welfare, which are both separate from the choice to neuter for the owner's benefit. While benefits of castration for cats are more conclusive, aside from population control, there is no definitive single recommendation for whether or not to neuter dogs, or at what age, and owners should make an educated decision after discussion with their veterinarian.

### Tail Docking (Amputation)

This procedure, amputating the distal portion of the tail, is “traditional” for certain breeds, such as hunting breeds and terriers. Reasons cited to have the tail docked include the potential to prevent injuries and to be able to more easily pull a terrier out of the ground when hunting (86). In some circumstances, the breeder will dock the tails, while other times a veterinarian will do it, and it is usually performed within a few days of birth. In Australia, the procedure is regulated and “…*should only be carried out in respect of those breeds with a known history or propensity to injury and/or damage in their tails in the course of their normal activities for therapeutic and/or prophylactic purposes…*” (87). Some states have also regulated this procedure (88). National veterinary medical associations also have position statements about cosmetic surgeries, including the statement from the AVMA which “…*opposes ear cropping and tail docking of dogs when done solely for cosmetic purposes. The AVMA encourages the elimination of ear cropping and tail docking from breed standards*” (89, 90). And one large veterinary corporation has banned the procedure for cosmetic reasons (91).

A survey conducted in Australia found that 75% of veterinarians believed that tail docking causes significant to severe pain, with none believing that dogs do not experience pain during this procedure. In contrast, roughly 80% of breeders believed that puppies experienced either mild or no pain (92). While there should be no argument that this procedure is painful, as part of the tail is amputated, it is uncertain for how long the pain lasts. One paper suggests that the pain experienced is relatively short-lived in dogs undergoing tail docking, and that, at the time of publication, only 10% of veterinarians used analgesics (93). While there are limited studies in dogs, there is evidence of behavioral and physiological responses to pain in production animals undergoing tail docking procedures (94). There are also behavioral effects from this procedure. Dogs showed more cautionary behaviors toward a short-tailed dog. The hypothesis is that it may be more difficult for the dog with the docked tail to signal appropriately to other dogs (95).

There is some evidence that dogs with intact tails are more likely to sustain injury to the tail compared to dogs whose tails were docked (96, 97). However, another study demonstrated that only three out of 2,000 visits to an emergency clinic in Australia were due to tail injuries; all three of these were due to post-docking complications (98). Currently, there is no evidence that pain due to tail injuries of dogs with complete tails is worse or more difficult to manage than the potential for chronic pain from tail docking. Therefore, one should weigh the pain from an unlikely injury against certain pain from tail docking.

There are flawed arguments in favor of tail docking in dogs. The most flawed one is in favor of tradition. Arguments in favor of tradition reflect an inherent sense of superiority of humans, and a disregard for the status of dogs in our society, especially as we have transformed from an agrarian to more urban society. If tail docking is done to prevent unlikely injuries incurred while hunting, we should ask the question: what is the likelihood for a particular dog to actually hunt? Given these weak arguments, there is little evidence to support the continued practice of tail docking.

### Ear Cropping (Cosmetic Otoplasty)

Ear cropping is a procedure in which the external pinna is reshaped to obtain an erect ear, usually performed when a dog is roughly 2–3 months of age. It is considered breed standard for some breeds, such as Doberman Pinschers and Great Danes. While breed clubs within the American Kennel Club (AKC) determine whether or not breed standard include cosmetic alterations via ear cropping or tail docking in order to show a dog in an AKC trial, owners of dogs with natural ears and tails report discrimination against them, as they are infrequently selected as winners (99, 100). Some states regulate ear cropping so that a licensed veterinarian must perform the procedure; Washington prohibits the procedure except when it is considered a customary husbandry practice (88). As with tail docking, some national veterinary medical associations have position statements against ear cropping in dogs, especially if done solely for cosmetic reasons (89, 90). As with tail docking, one large veterinary corporation has banned ear cropping for cosmetic reasons (91).

One erroneous argument made for ear cropping is to prevent illnesses and injuries in spaniels, terriers, other hunting dogs, and dogs with pendulous ears; however, there is no evidence to back up these claims (101). The etiology of otitis is related to many factors, including allergies, and breeds historically shown with cropped ears are no more likely to be diagnosed otitis (102–104). There should be no argument that this surgical procedure causes pain. As with tail docking, people will provide the argument in favor of “tradition” to support the continued practice of ear cropping. Given these weak arguments, there is little evidence to support ear cropping.

### Devocalization (Ventriculocordectomy)

Ventriculocordectomy is the partial or complete surgical removal of the vocal folds to reduce the volume of a dog's bark. Dogs still “bark,” and if not prevented from vocalizing post-op, are able to regain the ability to produce some sound. This surgical intervention does not address underlying reasons for barking, such as anxiety, social facilitation, aggression, and stereotypies, as well as being a more breed-typical behavior (105–107). Treating only the symptoms of any problem can actually increase behavioral and physiological stress, as evidenced in research performed on the use of anti-bark collars (108). The results of this study demonstrated that, while both electronic and citronella spray bark collars decreased barking, dogs wearing either collar had an increase in plasma cortisol. Another study demonstrated behavioral and physiological (salivary cortisol and heart rate) responses to an electronic shock collar (109). As with other elective procedures, veterinary organizations have published position statements and white papers on this subject. The AVMA policy states that “*Canine devocalization should only be performed by qualified, licensed veterinarians as a final alternative to euthanasia after behavioral modification to correct excessive vocalization has failed and after discussion of potential complications from the procedure with the owner*” and has a literature review supportive of this (110, 111). Some states passed various laws limiting or banning this procedure, including Massachusetts, Rhode Island, and Ohio (88). As with tail docking and ear cropping, one large veterinary corporation has banned devocalization (91).

Owner factors come into consideration when veterinarians are presented with a dog for devocalization. Until a bill was passed in 2012, landlords in California were allowed to require that a dog be devocalized as a condition of leasing (112). There may be pressure from neighbors to have their dog stop barking, sometimes resulting in tickets and fines from municipalities. Owners are faced at minimum with continued judgement and coercion, and at the gravest, pressure to relinquish or euthanize their pet. While treatment options exist for the underlying reasons for barking, some owners may find it difficult to adhere to the treatment recommendations. As such, the decision to devocalize or not turns into not only a welfare issue, but also an ethical dilemma, with devocalization balanced against potential relinquishment or euthanasia.

### Dental Procedures

Procedures range from vital pulpotomies, which is an endodontic procedure of capping only the canines or all of the teeth, to extracting only canine teeth, to extracting all of the teeth (113). Each of these procedures has the potential for a negative outcome, from failure of a crown resulting in a fractured tooth, to failure of a vital pulpotomy, resulting in an infected tooth root (114).

When these procedures are performed to “treat” aggression and prevent injury, it gives a false sense of security, as these dogs still can bite and cause crushing injuries. It does not eliminate the underlying motivation for aggression. If the animal is placed into a situation in which it feels that it must bite, its welfare is negatively impacted.

National veterinary organizations and specialty organizations have provided policy statements on such procedures. The AVMA is “*opposed to removal or reduction of healthy teeth in nonhuman primates and carnivores, except when required for medical treatment or approved scientific research*” (115). The American Veterinary Dental College supports the removal of crowns of teeth in selected cases where other corrective measures have failed, but also states that the removal of teeth will not prevent injury (116). As there is, at best, little safety benefit, weighed against the pain and other negative outcomes, to having these procedures done, they should not be performed.

### Declawing (Onychectomy)

Onychectomy is the surgical removal of the P3 phalynx, done to prevent the ability of a cat to damage the environment or injure people or other pets. Anecdotally it is estimated that up to 24% of cats in the United States are declawed (117). In a study on scratching behavior in cats, 52% of cat owners reported their cat to inappropriately scratch in the house; 65% of these cats reported their cat to inappropriately scratch at least daily. Respondents excluded from this study included those who owned declawed cats; of those owners, 45% chose to have their cat declawed to prevent damage, and 27% to prevent injury to people or other pets (118).

Feline rescue organizations and shelters historically have promoted the idea that declawed cats have a higher likelihood of being aggressive or eliminating inappropriately. Research into the circumstances surrounding declawed cats in shelters did not bear the same results (119). There were significantly fewer declawed cats in the shelter studied than would be expected based on the population at large. Where only 9% of the sheltered cats were declawed, it is estimated that 24% of the feline population at large at the time of the study were declawed. Additionally, there were fewer cats in the shelter reported to bite compared to population at large. Declawed cats also were no more likely to be euthanized at the shelter compared to cats that were not declawed; however, declawed cats stayed at the shelter longer. Owners surveyed after the procedure stated that they were generally satisfied or very satisfied with the procedure, and evidence of negative outcomes on behavior is limited (119–123). These numbers could be influenced by bias on the owners' part, as they elected to have their cats declawed.

It should come as no surprise that evidence shows declawing causes pain in cats, in part due to a number of surgical sequelae including retained bone fragments, osteomyelitis, excessive tissue handling during surgery, and complications from bandaging (124). While pain is an expected outcome of any surgery and should be ameliorated appropriately, there is evidence that there is short- and long-term pain due to onychectomy (124–129). Behaviors indicative of pain, such as forepaw shaking and laying on their side, were seen in cats that were declawed compared to those having a sham procedure performed (125). Although veterinarians provide pain control post-operatively, it does not seem to have a substantial immediate positive effect on a cat's gait; however, in one study (130), their gait was considered normal by 6 months (130–132). In another study, more cats were non- or limited-weight-bearing at discharge in control cats than in cats that received butorphanol post-operatively. By the second day, there were no differences between owner-assigned lameness scores (133).

Surgical methods and adherence to appropriate technique affect the outcome for declawing, as with all surgeries. A recent study demonstrated that 63% of cats had radiographic evidence of P3 remnants, and this was most highly correlated with back pain, periuria, and aggression (128). These results were seen far out from the surgery date. Other studies demonstrated that there were fewer negative welfare outcomes in cats in which a CO_2_ surgical laser was used (127, 134). Another study demonstrated that cats that underwent an onychectomy compared to a tendonectomy showed evidence of more pain and short-term behavioral changes (135).

Veterinarians have an evolving view on declawing. Two separate studies of veterinarians in Canada and the United States came to similar conclusions. Results included ~75% of veterinarians performed this procedure, most of them after counseling the owners about alternatives. Of these veterinarians, 60% of them performed the procedure less than monthly and between 10 and 15% performed it at least weekly (136, 137).

There are alternatives to declawing to prevent injury to people or other animals, or damage to the household. Physical alternatives include nail trims and nail covers (138). The benchmark of treatment includes behavior modification and environmental management, such as providing appropriate outlets for scratching and other species-typical behaviors and needs, preventing access to objects that one doesn't want scratched, and addressing the underlying reason for aggressive behavior (60, 118, 139–144).

As with procedures mentioned previously, some national veterinary organizations and other animal-related organizations have published policy statements on declawing (145, 146). The Canadian Veterinary Medical Association “*opposes elective and non-therapeutic Partial Digital Amputation (PDA), commonly known as declawing or onychectomy, of domestic cats*” (146). The AVMA published a detailed literature review that summarizes the pros and cons of this procedure, as well as outline alternatives (147). The Cat Fanciers' Association and The Canadian Cat Association do not allow declawed cats to be shown in sanctioned shows (148, 149). And most recently, three large veterinary corporations have banned elective declawing surgeries (150). Some municipalities, and even some countries, have banned this procedure (88, 151–153). Until a bill was passed in 2012, landlords in California were allowed to insist that a cat be declawed as a requirement for leasing (112).

Despite declawing performed not infrequently, and despite the research on this procedure producing equivocal results if performed correctly with appropriate pain control, there is no direct benefit to a cat to be declawed, other than potentially remaining in the household and not being relinquished or euthanized for problem scratching behavior.

## Summary

Veterinary medicine is more than the sum of its parts. It is not medical OR behavioral, it is treating an animal as a whole; these boundaries blur as we understand more their interrelationship. With this knowledge, it remains imperative for veterinarians to understand the potential behavioral consequences of surgery, and understand the underlying motivations for problem behaviors in pets. It is also incumbent amongst veterinary professionals to be champions for the welfare of the animal, and be able to communicate those points to owners and the public.

## Author Contributions

The author confirms being the sole contributor of this work and has approved it for publication.

## Conflict of Interest

The author declares that the research was conducted in the absence of any commercial or financial relationships that could be construed as a potential conflict of interest.

## Footnotes

^1^Available online at: https://championofmyheart.com/relaxation-protocol-mp3-files/
